# Response to trastuzumab-deruxtecan in metastatic triple-negative breast cancer with both HER2 mutation and low expression

**DOI:** 10.31744/einstein_journal/2026RC1689

**Published:** 2025-12-11

**Authors:** Caroline Dotto Amarger, Maria Isabel de Sampaio Rabello, Sofia Chaves Skaf, Fernando Moura, Janaína Pontes Batista Cassoli, Patrícia Taranto, Pedro Luiz Serrano Usón, Rafael Kaliks, Juliana Rodrigues Beal

**Affiliations:** 1 Hospital Israelita Albert Einstein Faculdade Israelita de Ciências da Saúde Albert Einstein São Paulo Brazil Faculdade Israelita de Ciências da Saúde Albert Einstein, Hospital Israelita Albert Einstein, São Paulo, Brazil.; 2 Hospital Israelita Albert Einstein Centro de Oncologia e Hematologia Einstein Família Dayan – Daycoval São Paulo SP Brazil Centro de Oncologia e Hematologia Einstein Família Dayan – Daycoval, Hospital Israelita Albert Einstein, São Paulo, SP, Brazil.; 3 Hospital Israelita Albert Einstein Centro de Medicina Personalizada São Paulo SP Brazil Centro de Medicina Personalizada, Hospital Israelita Albert Einstein, São Paulo, SP, Brazil.

**Keywords:** Breast neoplasms, Receptor, ErbB-2, Trastuzumab deruxtecan, Immunoconjugates, Precision medicine, Molecular targeted therapy

## Abstract

Breast cancer is one of the most prevalent and heterogeneous cancers worldwide; however, it remains a complex and enigmatic disease. Advances in targeted therapies combined with increasing knowledge about tumor subtypes and molecular profiling have placed precision medicine on the frontier of oncological treatments. The recent classification of certain HER2 tumors as HER2-low, along with emerging evidence from studies using antibody-drug *conjugates* to target specific tumor profiles, highlights the relevance of this case study. This report describes the treatment of a 72-year-old woman diagnosed with metastatic triple-negative breast cancer, classified as HER2-low with specific HER2 mutations. The patient showed disease progression despite standard treatments, leading to the use of trastuzumab deruxtecan (T-DXd), an antibody-drug conjugate targeting HER2, as a third-line therapy. Her positive response, which surpassed the median progression-free survival rates seen in clinical trials, highlights the potential of precision medicine, where treatment is customized based on genetic and molecular tumor profiles. These new therapeutic options for difficult-to-treat cancer subtypes expand treatment possibilities, ushering in a new era of personalized and precision-driven oncology.

## INTRODUCTION

Breast cancer is the most commonly diagnosed cancer among women worldwide, with an estimated 2.3 million new cases and 670,000 deaths by 2022.^( 1 )^ Considering the magnitude of its incidence, it is essential that screening, diagnosis, and treatment are available worldwide to improve the survival rates and quality of life of patients. Despite rapid scientific advances, many unanswered questions remain regarding the molecular mechanisms underlying the development of these tumors and the optimization of treatments for better outcomes. Breast carcinomas are highly heterogeneous diseases traditionally categorized into subgroups based on the immunohistochemical expression of estrogen receptor (ER), progesterone receptor (PR), and human epidermal growth factor receptor 2 (HER2). This classification is based on scientific literature, which has proven that different subgroups have various prognoses and respond distinctively to treatments.^( 2 )^ The first subcategory is hormone-positive tumors, which comprise approximately 70% of invasive breast cancers and are associated with the best overall survival rates and positive responses to endocrine therapy.^( 3 )^ The following subgroups are HER2-positive tumors, which exhibit increased cell proliferation rates and can either express ER or PR, with promising responses to HER2-targeted therapies.^( 4 )^ Lastly, the triple-negative (TNBC) cancers, which lack expression of any of the three aforementioned receptors, are the most aggressive and usually have worse survival rates and a high chance of recurrence.^( 2 )^ Given the diverse characteristics of various breast cancer subgroups that impact patient prognosis, there is currently no universal treatment protocol applicable to all cases. Consequently, oncological treatment options are increasingly tailored to individual patient needs to achieve optimal responses to therapy.

HER2 is an endothelial tyrosine kinase protein and an oncogene that stimulates cell proliferation and progression. Its activation occurs through homodimerization with another HER2 molecule or heterodimerization with other receptor monomers, such as HER1, HER3, or HER4,^( 4 )^ triggering downstream phosphorylation and signaling cascades that regulate gene expression involved in cell differentiation and survival. Approximately 20% of breast cancers present with ERBB2 amplification, which leads to HER2 overexpression and traditional eligibility for HER-2 targeted therapy.^( 5 )^ Until recently, HER2 status was considered positive if the tumor cells had a score of 3+ on the immunohistochemistry (IHC) assay or a score of 2+ combined with positive fluorescence *in situ* hybridization (FISH) molecular analysis.^( 5 )^ Recent developments have revealed greater diversity within tumors considered to be HER2 negative, showing that there are many different protein expression levels and introducing the concept of HER2-low tumors. These were defined as tumors with an IHC score of 1 or 2 and negative FISH results, indicating low HER2 expression without *ERBB2* amplification.^( 6 )^ Those that were once recognized as HER2 negative are now eligible for targeted HER2 therapy, such as antibody-drug conjugates (ADCs), thereby expanding the portfolio of anti-cancer treatment options for these types of tumors.^( 7 )^

In recent decades, advancements in targeted therapies have significantly transformed the clinical management of metastatic HER2-positive disease. A notable milestone occurred in 2001 when the combination of adjuvant trastuzumab, a humanized monoclonal antibody that inhibits HER2 homodimerization, with chemotherapy was linked to delayed disease progression and improved survival rates.^( 8 )^ This finding led to large-scale clinical trials in 2005, including the NCCTG N9831 and NSABP B-31 studies, in which the addition of trastuzumab to paclitaxel following a regimen of doxorubicin and cyclophosphamide substantially reduced the recurrence and mortality in women with HER2-positive breast cancer.^( 9 , 10 )^ In the following years, a monoclonal antibody that inhibits HER2 heterodimerization, called pertuzumab, was evaluated in patients whose disease progressed despite prior trastuzumab therapy. When combined with trastuzumab, pertuzumab was effective and encouraged patient response.^( 11 )^ However, studies have shown that anti-HER2 therapy is only effective in patients with an IHC score of 3+ or 2+ with a positive FISH assessment, indicating that patients classified as HER2-low would not benefit from these treatments.^( 12 )^

Clinical trials and ongoing research have led to the development of targeted therapies, including tyrosine kinase inhibitors and antibody-drug conjugates (ADCs), in a new era of precision oncology. Trastuzumab deruxtecan (T-DXd) is an ADC that binds an anti-HER2 monoclonal antibody to a cytotoxic agent through a cleavable tetrapeptide-based linker, creating a dual-action mechanism that combines targeted therapy with chemotherapeutic cytotoxicity.^( 13 )^ This drug complex works by releasing the deruxtecan payload into tumor cells, inducing DNA damage and apoptosis. Unlike many anti-HER2 therapies that require HER2 overexpression for efficacy, T-DXd remains effective in HER2-low tumors because of its membrane-permeable and highly potent payload, high drug-to-antibody ratio (8:1), and bystander effect on neighboring tumor cells.^( 13 )^

The first approval by the U.S. Food and Drug Administration (FDA) was granted in 2019 following the Destiny-Breast01 trial, which included 184 patients with unresectable and/or metastatic HER2-positive breast cancer and demonstrated promising response rates.^( 14 )^ Building on these findings, the Destiny-Breast 04 trial was conducted to evaluate the positive outcomes of using T-DXd in patients with low HER2 expression. The results showed longer progression-free and overall survival with TDXd than with the physician's choice of chemotherapy.^( 7 )^ Subsequently, researchers performed the Destiny-Pan Tumor02 trial to assess the efficacy of T-DXd in patients with HER2-expressing tumors, irrespective of the tumor site of origin. The study confirmed significant clinical benefits and survival outcomes, reinforcing the concept of T-DXd as a tumor-agnostic therapy that targets cancers based on molecular features rather than the tissue of origin.^( 15 )^

Besides treating cancers with varying degrees of HER2 expression, researchers have questioned whether T-DXd is active against tumors harboring mutant HER2 proteins. In 2022, the Destiny-Lung01 trial showed lasting anticancer activity in patients with non-small-cell lung cancer (NSCLC) refractory to standard treatments.^( 16 )^ After this clinical advancement, scientists explored the possibility of T-DXd being active against other types of unresectable and/or metastatic solid cancers with HER2 mutations, leading to the development of the Destiny-Pan Tumor01 trial. Completed in 2023, the study showed sustained results, with over 50% of the patients remaining free of progression after 18 months of treatment. Notably, breast cancer has emerged as a tumor type with particularly favorable outcomes following ADC therapy.^( 17 )^

This case study describes the exceptional and durable response of a patient with triple-negative metastatic breast cancer harboring both low HER2 expression and a HER2-activating mutation to T-DXd, used as third-line therapy. Although HER2-low and HER2-mutant tumors are increasingly recognized as therapeutic targets, evidence regarding their combined presence and clinical implications remains limited, particularly in triple-negative breast cancer. This report highlights the importance of integrating molecular profiling into clinical decision-making and supports the rationale for expanding the use of HER2-targeted therapies beyond traditional HER2-positive classifications.^( 18 )^ The aim of the study was to help guide future research and contribute to the growing field of precision oncology, especially in breast cancer management.

## CASE REPORT

A 72-year-old patient with a history of breast cancer at 35 years of age and a negative germline next generation sequence panel was diagnosed with metastatic breast cancer in October 2021. Breast biopsy revealed a triple-negative invasive lobular carcinoma with pleomorphic features. Immunohistochemistry (IHC) for HER2 revealed dubious staining, although fluorescence *in situ* hybridization (FISH) was negative.

**Figure 1:**
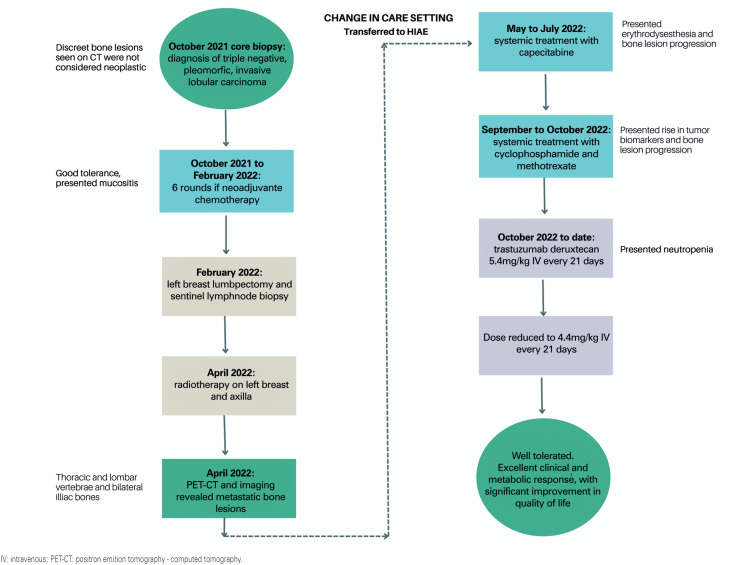
Flow chart of patient's treatment and responses

IV: intravenous; PET-CT: positron emition tomography - computed tomography.

At an external facility where she was initially treated, bone lesions were initially not considered metastatic, leading to a curative-intent treatment approach: neoadjuvant chemotherapy was used to shrink the tumor before surgery, breast-conserving surgery with sentinel lymph node biopsy, and adjuvant radiation therapy. The first follow-up PET/CT scan in April 2022 revealed residual cancer in the breast, and the iliac bone lesions became lytic, which is a marker of response to treatment. At this point, the patient was transferred to *Hospital Israelita Albert Einstein* (HIAE), where she started systemic first-line treatment using capecitabine with the intent to treat the metastatic disease. She developed palmar-plantar erythrodysesthesia and progression of the bone lesions; therefore, she was administered second-line therapy with cyclophosphamide 50 mg/day and methotrexate 2×2,5mg/day. The metastatic bone lesions continued to progress, and she showed laboratory evidence of progression with an increase in tumor biomarkers, necessitating a new line of systemic treatment ( Figure 1 ).

Although her germline panel was negative, considering the characteristics of this triple-negative neoplasm, the patient's medical history, and recent scientific literature supporting the benefit of targeted therapies in tumors with somatic mutations, a somatic next generation sequence panel was requested.

The tumor harbored two mutations in ERBB2, which encodes HER2. These activating mutations respond well to targeted medications because the receptors tend to remain in an open conformation. Mutations V842I and L755S confer hyperactivation of the PI3K and MAPK signaling pathways, favoring cell survival.^( 16 )^ Although these mutations are associated with resistance to tyrosine kinase inhibitors, recent studies have shown that these cells are also susceptible to ADCs. Unlike tyrosine kinase inhibitors, which primarily inhibit signaling pathways, ADCs operate via receptor internalization. Due to activating mutations, these receptors remain accessible, thereby enhancing the efficacy of the drug. ^( 17 )^

This case was discussed by a multidisciplinary molecular tumor board, with T-DXd as the next treatment option for metastatic HER2-low triple-negative breast cancer. Associating this conclusion with scientific studies that support the use of this drug in HER2-mutated tumors, the medical team chose to initiate trastuzumab deruxtecan at 5.4mg/kg IV on Day 1 every 21 d until disease progression or the toxicity limit was reached ( Figure 1 ).

As of October 28, 2022, the patient received T-DXd with good tolerability. She was initially prescribed 5.4mg/kg every 21 d; however, the dose was reduced to 4.4mg/kg due to the presence of neutropenia. Follow-up imaging revealed no pulmonary toxicity or a consistent complete metabolic response.

## CONCLUSION

This case report describes an elderly patient with triple-negative metastatic breast cancer, classified as HER2-low with HER2 mutations, who exhibited a remarkable and sustained response to T-DXd as third-line therapy. The current duration of the positive response has reached 20 months, surpassing the median progression-free survival of 10.1 months in the DESTINY-Breast04 trial and 6.9 months in the DESTINY-PanTumor02 trial. This study demonstrates the potential of tailored treatments that address specific tumor profiles, offering new hope for difficult-to-treat cancers.

Further research is required to determine which HER2 mutations and histological subtypes are more sensitive to T-DXd, as well as to other types of biomarker-guided therapies. Case reports and prospective studies should be conducted and discussed to review future patient-specific treatment options for breast and other cancers.

The principles of precision medicine – prevention, participation, personalization, and prediction – comprise the future of oncological treatment. This evolving medical approach enables healthcare workers to make more accurate diagnoses and offer individualized therapies. Scientific advances are constantly proving the benefits of personalized medical decisions, which consider the differences in disease subtypes as well as patients’ lifestyles, environments, and genetics. All these pillars guided the management of this case and helped explain the extraordinary results obtained.

## DATA AVAILABILITY

The underlying content is contained within the manuscript.
